# Tilting at Windmills: Why Attacks on Repression Are Misguided

**DOI:** 10.1177/1745691620927674

**Published:** 2020-08-11

**Authors:** Chris R. Brewin

**Affiliations:** Clinical Educational and Health Psychology, University College London

**Keywords:** memory, trauma, repression

## Abstract

In the November 2019 issue of *Perspectives*, Otgaar et al. argued that the “memory wars” persist and that “the controversial issue of repressed memories is alive and well and may even be on the rise” (p. 1072). Their thesis overlooked the well-established consensus that recovered memories of trauma may be genuine, false, or a mixture of the two and instead focused on a disputed mechanism: unconscious repression. A formal cocitation analysis identified the major publications mentioning repressed memories, but none endorsed a theory of unconscious repression. Studies of beliefs about repressed memories by the general public and other groups do not support Otgaar et al.’s thesis either because these studies did not adequately assess the key ideas defining the theory of repression. Clinical evidence is consistent with recovered memories occurring in many different forms of therapy, including ones that do not use suggestive techniques or rely on the concept of repression. Thus, Otgaar et al. have proposed the existence of a problem for which little objective evidence can be found. Continuing theoretical uncertainties about the mechanisms responsible for forgetting are less important than the general recognition since the 1990s that suggestive therapy and attempts to exhume memories are hazardous and generally inappropriate.

The “memory wars” were prompted by very real concerns about inappropriate therapeutic practice. Some clinicians believed the symptoms experienced by certain patients might be caused by sexual abuse in their childhood that they had forgotten and that it would be beneficial to help them recover the memory of what had happened. The conflict as such was between (a) those who appeared to believe the content of such recovered memories could always be relied on (although to my knowledge no article published in a reputable scientific or clinical journal ever claimed this) and (b) those who did not believe such traumatic experiences could be forgotten and so they must of necessity be of iatrogenic origin and false. Independent reviewers and professional organizations swiftly considered the relevant evidence, including the types of traumatic events that were reported as being recovered, whether this occurred inside or outside of therapy, and the amount of corroboration available. The general conclusion, which still holds today, was that recovered memories may be true, false, or a mixture of the two and that the use of suggestive therapeutic techniques with patients who do not remember any history of abuse is hazardous and generally inappropriate (Belli, 2012; Goodman-Delahunty, Nolan, & Van Gijn-Grosvenor, 2017; Lindsay & Briere, 1997; Lindsay & Read, 1995; McNally & Geraerts, 2009; Wright, Ost, & French, 2006).

Despite their argument that the memory wars continue and may be getting worse, Otgaar et al. (2019) do nothing to suggest this agreement has broken down. They describe some authors as being satisfied with the general acceptance that it is wrong to believe in the content of such memories without reservation, whereas others are described as pursuing an understanding of what mechanisms might account for those instances of true recovered memories, consistent with the above consensus. Otgaar et al., however, have projected onto these reasonable positions a continuing conflict about an underlying mechanism: repression (subsequently extended to include dissociative amnesia). They follow Loftus (1993) in pursuing a skeptical argument focused on undermining the theoretical mechanism by which traumatic events might come to be forgotten. I argue that, like Don Quixote and his misguided urge to attack the giants that turned out to be windmills, Otgaar et al. are engaging with an imaginary enemy.

The point that issues of mechanism (i.e., repression) are logically distinct from the observations they are designed to explain (i.e., the recovery of memories that the person says they had previously forgotten) has been made many times (Brewin & Andrews, 1998; Lindsay & Briere, 1997). A variety of theoretical mechanisms are available that could potentially explain such recovery, including cueing by aspects of the internal or external environment, metacognitive failures, reinterpretation of events in light of new knowledge, and the release of inhibition brought about by deliberate suppression or intentional forgetting (Anderson & Hanslmayr, 2014; Brewin, 2012; Brewin & Andrews, 1998; McNally & Geraerts, 2009; Roediger & Bergman, 1998; Schooler, Ambadar, & Bendiksen, 1997). Clinicians commonly refer to memories simply as being *forgotten* (i.e., lost both from semantic and episodic memory) and then subsequently recovered—such memory recovery occurs regularly, involves a wide variety of events (both traumatic and nontraumatic), occurs in therapeutic and nontherapeutic situations, and is often corroborated (Andrews et al., 1999; Brewin & Andrews, 1998; Gleaves, Smith, Butler, & Spiegel, 2004; Read & Lindsay, 2000).

In this article, I therefore test a number of basic assumptions implied by Otgaar et al. Having established how repression has been defined in this context, I conduct a formal bibliometric analysis to test which publications mentioning repression are most often cocited together with other publications in the field. I then inspect these publications to see whether they use the term *repression* in the sense identified by the skeptics as problematic. Second, I consider whether the survey data cited by Otgaar et al. in fact support widespread belief in such a version of repression. Third, I consider the evidence that recovered memories are common in clinical settings, occur during therapy sessions, and are associated with therapists who have a theoretical orientation that endorses repression. I close by discussing the potential negative consequences of the continuing attacks on repression.

## What Is Repression?

The article that originally drew attention to the possibility of false memories of childhood sexual abuse (Loftus, 1993) claimed that the forgetting of traumatic events such as child sexual abuse was justified by clinicians in terms of repression, a psychoanalytic concept. As stated in several publications by skeptics and recapitulated by Otgaar et al. (2019), “the notion of repressed memories encompasses three ideas: People repress traumatic experiences, the repressed content has psychopathological potential, and recovering traumatic content is necessary for engendering symptom relief” (p. 1073).

Despite awareness that the term repression was sometimes used by Freud to refer to a conscious process that we would now term intentional or motivated forgetting (Erdelyi, 1990; Loftus & Ketcham, 1994), repression has been repeatedly treated by skeptics of recovered memory as though it mainly or exclusively refers to an unconscious process (Lynn, Evans, Laurence, & Lilienfeld, 2015; Otgaar et al., 2019; Patihis, Lilienfeld, Ho, & Loftus, 2014; Piper, Lillevik, & Kritzer, 2008). For example, skeptics frequently cite research demonstrating a lack of empirical support for repression (Holmes, 1990) but often fail to mention that this work is relevant only to the unconscious meaning of the term. Thus, as used by skeptics, the definition of repression contains at least two distinct elements, one (theoretical) being an explanation of forgetting trauma in terms of an unconscious process and one (practical) being concerned with clinical strategies.

## Who Refers to Repression as a Theoretical Construct?

A recent bibliometric analysis of articles on the recovered-memory controversy published in the 21st century (Dodier, 2019) distinguished between articles that were skeptical of repression and those that were “sympathetic” to it. How this sympathy was defined or measured was not stated. “Repressed memory” or “repressed memories” were represented as keywords on only about 17% of the 145 articles reviewed by Dodier. An inspection of the articles cited as examples of those sympathetic to repression revealed that none in fact endorsed an unconscious version of the process or approved of exhuming forgotten memories but instead discussed a variety of mechanisms that could underlie forgetting (Brand, Collins, & McEwen, 2018; Brewin, 2007; Dalenberg et al., 2012; Freyd, DePrince, & Gleaves, 2007; Williams, 1994). There were substantially more publications by repression skeptics than by those who were supposedly sympathetic to repression.

Another recognized bibliometric approach to discerning the structure of intellectual knowledge in a given area is cocitation analysis. Two sources are co-cited if both appear in the reference list of a third document, and the number of times this happens is defined as cocitation strength. This measure reflects the degree of relationship or association between publications as perceived by the population of citing authors (Osareh, 1996). When the same pairs of sources are cocited multiple times, clusters of research begin to form that share a common theme. Cocitation relationships can be mapped, giving a visual representation of the elements and how they are associated. The analysis can identify the most important sources endorsing the concept of repression and quantify the extent to which they are cocited with different clusters of research on recovered and traumatic memory.

The Web of Science Core Collection, MEDLINE, and Current Contents Connect databases were searched on January 2, 2020, specifying articles published between 1993 and 2020 and that included any of the following terms: “recovered memor*” OR “memory recovery” OR “recovery of memor*” OR “repressed memor*” OR “memory repression” OR “repression of memor*.” This yielded 537 articles; of these, 102 were excluded because they dealt with unrelated topics (e.g., engineering), 35 were excluded because they were published in journals unrelated to psychology or mental health (e.g., English literature), 202 were excluded because they were not full articles with comprehensive reference lists (e.g., commentaries, letters), and 10 were excluded because they were published in a foreign language. This left 174 articles from the Web of Science Core Collection. Similar searches produced eight extra articles from MEDLINE and six from Current Contents Connect.

The number of times these articles were cited in the Web of Science Core Collection database was strongly positively skewed, with a median of 7 and a range of 0 to 743. Altogether they referenced 4,886 separate books and articles, with citations to these sources also being strongly positively skewed. To limit the books and articles to the most influential ones in the field while taking into account the low average citation rate, those cited at least eight times in this secondary data set were selected for analysis (producing 93 books and articles).

**Fig. 1.**
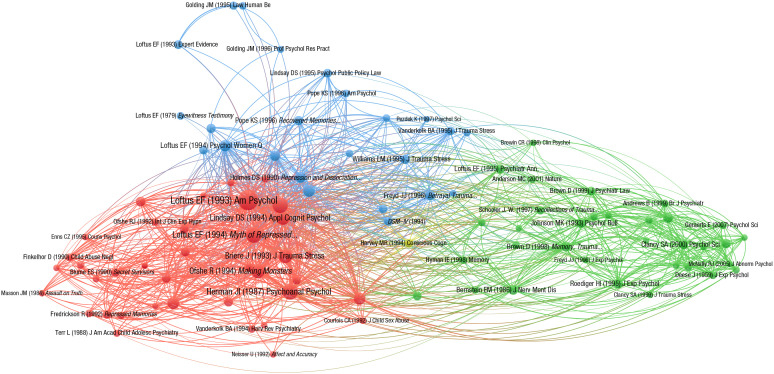
Cocitation analysis of articles and books on repressed and recovered memories. The distance between two items reflects the strength of the relation (number of cocitations) between the items. A smaller distance generally indicates a stronger relation. The more important an item, the larger its label and its circle. Each item’s circle is displayed in the color corresponding to the cluster to which it was assigned. To avoid overlapping labels, only a subset of all labels is displayed.

Cocitation relations between these books and articles, expressed as a co-occurrence matrix, were mapped using the visualization of similarities (VOS) mapping technique (van Eck & Waltman, 2007). The resulting distance-based map (Fig. 1) was produced using the VosViewer program (van Eck & Waltman, 2010). The program output includes the number of items with which a given item shares cocitation links and the total link strength, reflecting the number and strength of the links.

The analysis resulted in three substantial clusters. Articles and books constituting the clusters are listed in Table 1 and are represented visually in Figure 1 by a label and a circle. The first (red) cluster consisted of the most important items (gauged by their number of links and total link strength) and included seminal articles and books that raised concerns about repressed memories in clinical practice in the early 1990s (Loftus, 1993; Loftus & Ketcham, 1994; Ofshe & Watters, 1994), along with some of the key articles documenting experiences relevant to the forgetting and recovery of traumatic memories (Briere & Conte, 1993; Herman & Schatzow, 1987; Williams, 1994). The second (green) cluster consisted mainly of books and articles addressing issues related to false memories either experimentally or theoretically (e.g., Clancy, Schacter, McNally, & Pitman, 2000; Hyman & Billings, 1998; Johnson, Hashtroudi, & Lindsay, 1993; McNally, Clancy, & Schacter, 2001; Schooler et al., 1997). The third (blue) cluster consisted mainly of articles reporting clinical studies and surveys of the experience of traumatic or recovered memories (Elliott & Briere, 1995; Polusny & Follette, 1996; Poole, Lindsay, Memon, & Bull, 1995).

**Table 1.** Articles and Books Constituting the Three-Cluster Solution

Cluster 1 (38 items)	Cluster 2 (33 items)	Cluster 3 (21 items)
Andrews et al. (1995)	Anderson & Green (2001)	American Psychiatric Association (1994)
Bass & Davis (1988)	Andrews et al. (1999)	Dalenberg (1996)
Beitchman et al. (1992)	Bernstein & Putnam (1986)	Elliott & Briere (1995)
Blume (1990)	Brewin & Andrews (1998)	Freyd (1996)
Briere & Conte (1993)	Brown et al. (1998)	Golding et al. (1995)
Ceci et al. (1994)	Brown et al. (1999)	Golding et al. (1996)
Courtois (1988)	Ceci & Loftus (1994)	Holmes (1990)
Courtois (1992)	Clancy (2005)	Key et al. (1996)
Della Femina et al. (1990)	Clancy, McNally, & Schacter (1999)	Lief & Fetkewicz (1995)
Enns et al. (1995)	Clancy et al. (2000)	Lindsay & Read (1995)
Feldman-Summers & Pope (1994)	Crews (1995)	Loftus (1979)
Finkelhor et al. (1990)	Deese (1959)	Loftus et al. (1993)
Fredrickson (1992)	Freyd & Gleaves (1996)	Pezdek et al. (1997)
Gold et al. (1994)	Garry et al. (1996)	Polusny & Follette (1996)
Herman & Schatzow (1987)	Geraerts et al. (2005)	Poole et al. (1995)
Herman (1981)	Geraerts et al. (2006)	K. S. Pope (1996)
Herman (1992)	Geraerts et al. (2007)	K. S. Pope & Brown (1996)
Laurence & Perry (1983)	Geraerts et al. (2009)	Schacter (1996)
Lindsay & Read (1994)	Hyman et al. (1995)	van der Kolk & Fisler (1995)
Loftus (1993)	Hyman & Billings (1998)	Williams (1995)
Loftus, Garry, et al. (1994)	Johnson et al. (1993)	Yapko (1994a)
Loftus & Ketcham (1994)	Loftus & Pickrell (1995)	
Loftus, Polonsky, et al. (1994)	McNally (2003)	
Masson (1984)	McNally et al. (2004)	
Neisser & Harsch (1992)	McNally et al. (2005)	
Ofshe (1992)	McNally, Clancy, & Schacter (2001)	
Ofshe & Watters (1994)	McNally, Clancy, Schacter, & Pitman (2000)	
Pendergrast (1995)	McNally & Geraerts (2009)	
H. G. Pope & Hudson (1995)	Myers et al. (1998)	
Pynoos & Nader (1989)	Porter et al. (1999)	
Spence (1982)	Roediger & McDermott (1995)	
Terr (1988)	Schooler, Ambadar, & Bendiksen (1997)	
Terr (1991)	Schooler, Bendiksen, & Ambadar (1997)	
Terr (1994)		
van der Kolk (1994)		
Wakefield & Underwager (1992)		
Williams (1994)		
Yapko (1994b)		

Note: A fourth cluster consisted of only one item: Harvey and Herman (1994).

Most of the authors whose work has been criticized by memory skeptics as promoting the concept of repression or advocating memory recovery (Bass & Davis, 1988; Blume, 1990; Fredrickson, 1992; Freyd, 1996; Herman & Schatzow, 1987; Terr, 1991) featured in the red cluster. Their number of links to articles and books in all three clusters and total link strength are shown in Table 2. The most cocited items are by Herman and Schatzow (1987), followed by Terr (1991) and Bass and Davis (1988). No additional items that endorsed repression and had high link strength were identified by the analysis. These conclusions were not altered by choosing different thresholds for including citations from the secondary data set.

**Table 2.** Cocitation of Sources Cited by Skeptics as Endorsing Repression

Source	Total link strength	Total links	Number of citations
Bass & Davis (1988)	368	80	29
Blume (1990)	213	62	14
Fredrickson (1992)	173	56	13
Freyd (1996)	238	80	18
Herman & Schatzow (1987)	563	92	45
Terr (1991)	388	87	27

In a final step, the sources listed in Table 2 were inspected to determine what version of repression they described. None appeared to specifically endorse the unconscious version of repression about which skeptics have raised concerns. Likewise, another source in the blue cluster that referred to repression (Brown, Scheflin, & Whitfield, 1999) discussed it in the context of various mechanisms and did not specifically endorse either the conscious or unconscious use of the term.

## Surveys of Beliefs About Repression

Another major plank in the effort by Otgaar et al. to persuade the reader of continuing hostilities consisted of claims that not only the general public but also mental health and legal professionals have mistaken beliefs about memory that are at odds with scientific evidence. Conclusions have typically been based on the fact that large numbers agree with a single questionnaire item such as “Traumatic experiences can be repressed for many years and then recovered” (Kassin, Tubb, Hosch, & Memon, 2001). This item does not specifically identify the unconscious form of repression that remains unsupported by empirical evidence (Brewin & Andrews, 2014). It also does not address the other critical aspects of repression identified by skeptics (i.e., that the repressed content has psychopathological potential and that recovering traumatic content is necessary for engendering symptom relief). Perhaps most importantly, it does not throw light on people’s understanding of the term repression and whether this term is anything more than a synonym for motivated forgetting (Anderson & Hanslmayr, 2014).

In a recent survey, Brewin, Li, Ntarantana, Unsworth, and McNeilis (2019) supplemented the original repression item of Kassin et al. (2001) with an alternative: “Traumatic experiences can be deliberately blocked out for many years and then recovered.” Respondents agreed with this item at the same rate as they did with the original item. There are several possible explanations of this finding: Respondents may specifically endorse a belief in conscious repression, may endorse both conscious and unconscious repression, or may not feel equipped to make a meaningful distinction between the two. What is clear is that conclusions frequently advanced in the literature (Benton, Ross, Bradshaw, Thomas, & Bradshaw, 2006; Lynn et al., 2015; Melinder & Magnussen, 2015; Patihis, Ho, Tingen, Lilienfeld, & Loftus, 2014) concerning the public’s beliefs in unconscious repression are as yet unwarranted.

## Repressed Memories and Clinical Strategies

As noted by Otgaar et al., recent articles have claimed that problematic practices involving repressed memories are still prevalent today. In a large general population survey, Patihis and Pendergrast (2019) reported that, after adjustment, 7% of their total sample reported seeing therapists who discussed the possibility of repressed abuse, and 5% reported recovering memories of abuse in therapy for which they had no previous memory. Both were more likely to have happened in the 1990s than in subsequent years. These results are difficult to interpret because the first question did not ask who raised the issue of repressed memory, the therapist or the patient, or in what context, and whether the discussion preceded or followed any actual memory recovery. Indeed, it is not clear whether the therapist or respondent used the actual terms *repression* or *repressed*, as alternatives were not suggested in the survey. In response to their second question, 42% of those recovering memories mentioned sexual abuse, but 74% mentioned emotional abuse and 51% mentioned physical abuse. Memories were recovered in many different types of therapy, including cognitive-behavior therapy. Approximately 30% reported remembering the abuse during a therapy session and 30% outside a therapy session; the remaining 40% reported that memories returned both during and outside a therapy session.

A replication was conducted in France (Dodier, Patihis, & Payoux, 2019), which, as the authors noted, has a stronger psychoanalytic tradition than the United States. Despite this tradition, only 4.4% of a general population sample reported seeing therapists who discussed the possibility of repressed abuse; the highest rate occurred between 1995 and 1999 and the lowest rate occurred between 2015 and 2018. Moreover, 2.5% reported recovering memories of abuse in therapy for which they had no previous memory. In this sample, recovered memories of sexual abuse were the most common, although memories of physical and emotional abuse also featured regularly. The type of therapy in which memory recovery was most likely to occur was behavior therapy (a form of therapy that does not include a concept of repression and traditionally does not dwell on childhood experience). Participants were more likely than their therapists to first broach the topic of recovered memory and reported recovered memories significantly more often when they first addressed the issue of repressed memories than when it was the therapist who first mentioned it, consistent with previous suggestions (Brewin & Andrews, 2017).

The findings of both studies were in line with previous research (Andrews et al., 1995, 1999) showing that memory recovery is a common therapeutic experience that usually cannot be explained through appeals to therapeutic suggestion or “recovered memory therapy.” In the absence of any evidence that recovered memories were likely to be false, as Patihis and Pendergrast (2019) suggested they were, the most parsimonious explanation is that many unpleasant experiences are in fact forgotten and that therapy creates an opportunity for these experiences to come to mind.

## Conclusion: The Downside of Attacking Repression

Several conclusions are evident from the literature reviewed above. It is widely accepted that traumatic events can sometimes be completely forgotten and then remembered later, although there is little understanding of why this occurs. Clinical suggestions about candidate mechanisms have been poorly defined, and it is unclear how mechanisms more firmly grounded in cognitive psychology map onto the clinical data. No source has been identified that argued in favor of the unconscious form of repression as an explanation for forgetting. References to repression, whether by lay people or mental health professionals, are likely to be little more than an attempt to re-label observations of forgetting trauma. With regard to the other key idea about the concept of repression identified by skeptics—that such memories have to be actively recovered in therapy—I found no source published after the early 1990s that supported this idea.

Also contrary to Otgaar et al.’s assertions is evidence that mainstream psychotherapists and clinical psychologists report being more cautious about recovering repressed memories today than they were 20 years ago (Patihis, Ho, et al., 2014), and this is supported by the reports of their clients (Dodier et al., 2019; Patihis & Pendergrast, 2019). The data, including those produced by skeptics themselves, show that recovered memories of traumatic events continue to be observed inside and outside clinical settings and involve a variety of events, and occur in a variety of different contexts. There appears to be no association with psychoanalytic therapy, the form of treatment most closely associated with the concept of repression, and no evidence that therapists are systematically engaging in inappropriate suggestive therapy (although individual examples of bad or ill-informed practice undoubtedly occur and surface from time to time in the courts).

All of this is incompatible with the claim made by Otgaar et al. (2019) that “the controversial issue of repressed memories is alive and well and may even be on the rise” (p. 1072). Nothing has happened to disturb the professional consensus on recovered memories first put forward in the 1990s and the improvements in practice that followed. Theoretical issues remain unresolved but are unimportant compared with the need for changes in practice that, having been generally accepted, were rapidly put into effect by professional bodies and recognized clinical-training courses.

Are there any dangers attached to attempting to prolong a conflict that existed for only a short time? One concern is that keeping the narrative focused on unconscious repression or dissociative amnesia rather than the more neutral concept of forgetting may have the effect of discrediting the validity of genuine recovered memories of sexual trauma. Instead of presenting the scientific and professional consensus that traumatic events can sometimes be forgotten and later remembered (for reasons that are not well understood), the courts’ attention is drawn to disputed concepts as though these concepts provide the only recognized explanation for memory recovery.

By primarily appearing to blame therapists for using suggestive therapy, Otgaar et al. also deflect from consideration those cases in which clients have convinced themselves for whatever reason that they have had abusive experiences in the absence of any conscious memory of them. Here the problem is not so much the therapist setting out with an inappropriate treatment but the failure to educate clients that even highly emotional images that spontaneously come to mind may not correspond to actual events (Brewin & Andrews, 2017).

In seeking to keep an old conflict alive, Otgaar et al. create division rather than finding solutions. The important issue is why and how traumatic events can sometimes come to be forgotten. This requires open-minded inquiry that recognizes the complexities of people’s lives (particularly those of children exposed to severe adversity); a developmental perspective on coping, memory, and attachment; and the willingness to consider multiple scenarios and theoretical possibilities. It is time to resurrect the spirit of the 1996 NATO Advanced Studies Institute conference on the recollection of trauma (Lindsay & Briere, 1997), at which scientists and therapists pledged to work collaboratively to build a future based less on rhetoric and more on reliable evidence.

## Supplemental Material

Brewin_Supplemental_Material – Supplemental material for Tilting at Windmills: Why Attacks on Repression Are Misguided Click here for additional data file. Supplemental material, Brewin_Supplemental_Material for Tilting at Windmills: Why Attacks on Repression Are Misguided by Chris R. Brewin in Perspectives on Psychological Science

## Transparency

*Action Editor:* Laura A. King

*Editor:* Laura A. King
