# Supplementary material for: Tilting at Windmills: Why Attacks on Repression Are Misguided
Source: Perspect Psychol Sci. 2020 Aug 11;16(2):443–53. doi: 10.1177/1745691620927674 (PMC7961625; doi:10.1177/1745691620927674)
Supplement: Brewin_Supplemental_Material – Supplemental material for Tilting at Windmills: Why Attacks on Repression Are Misguided [file Brewin_Supplemental_Material.pdf]

Supplemental Material

Articles and books included in the co-citation analysis

American Psychiatric Association. (1994). *Diagnostic and statistical manual of mental disorders (4th ed.)*. Washington, DC: American Psychiatric Association.

Anderson, M. C., & Green, C. (2001). Suppressing unwanted memories by executive control. *Nature, 410*, 366-369. doi:10.1038/35066572

Andrews, B., Brewin, C. R., Ochera, J., Morton, J., Bekerian, D. A., Davies, G. M., & Mollon, P. (1999). Characteristics, context and consequences of memory recovery among adults in therapy. *British Journal of Psychiatry, 175*, 141-146. doi:10.1192/bjp.175.2.141

Andrews, B., Morton, J., Bekerian, D. A., Brewin, C. R., Davies, G. M., & Mollon, P. (1995). The recovery of memories in clinical practice - Experiences and beliefs of British Psychological Society practitioners. *The Psychologist, 8*, 209-214.

Bass, E., & Davis, L. (1988). *The courage to heal: A guide for women survivors of child sexual abuse*: New York: Harper & Row.

Beitchman, J. H., Zucker, K. J., Hood, J. E., Dacosta, G. A., Akman, D., & Cassavia, E. (1992). A review of the long-term effects of child sexual abuse. *Child Abuse & Neglect, 16*, 101-118. doi:10.1016/0145-2134(92)90011-f

Bernstein, E. M., & Putnam, F. W. (1986). Development, reliability, and validity of a dissociation scale. *Journal of Nervous and Mental Disease, 174*, 727-735. doi:10.1097/00005053-198612000-00004

Blume, E. S. (1990). *Secret survivors: Uncovering incest and its aftereffects in women*: New York: Wiley.

Brewin, C. R., & Andrews, B. (1998). Recovered memories of trauma: Phenomenology and cognitive mechanisms. *Clinical Psychology Review, 18*, 949-970. doi:10.1016/s0272-7358(98)00040-3

Briere, J., & Conte, J. (1993). Self-reported amnesia for abuse in adults molested as children. *Journal of Traumatic Stress, 6*, 21-31. doi:10.1002/jts.2490060104

Brown, D., Scheflin, A. W., & Hammond, D. C. (1998). *Memory, trauma treatment, and the law*: New York: WW Norton.

Brown, D., Scheflin, A. W., & Whitfield, C. L. (1999). Recovered memories: The weight of the evidence in science and in the courts. *Journal of Psychiatry and Law, 27*, 5-156. doi:10.1177/009318539902700102

Ceci, S. J., & Loftus, E. F. (1994). Memory work - A royal road to false memories. *Applied Cognitive Psychology, 8*, 351-364. doi:10.1002/acp.2350080405

Ceci, S. J., Loftus, E. F., Leichtman, M. D., & Bruck, M. (1994). The possible role of source misattributions in the creation of false beliefs among preschoolers. *International Journal of Clinical and Experimental Hypnosis, 42*, 304-320. doi:10.1080/00207149408409361

Clancy, S. A. (2005). *Abducted: How people come to believe they were kidnapped by aliens*: Cambridge, MA: Harvard University Press.

Clancy, S. A., McNally, R. J., & Schacter, D. L. (1999). Effects of guided imagery on memory distortion in women reporting recovered memories of childhood sexual abuse. *Journal of Traumatic Stress, 12*, 559-569. doi:10.1023/a:1024704815234

Clancy, S. A., Schacter, D. L., McNally, R. J., & Pitman, R. K. (2000). False recognition in women reporting recovered memories of sexual abuse. *Psychological Science, 11*, 26-31. doi:10.1111/1467-9280.00210

Courtois, C. A. (1988). *Healing the incest wound: Adult survivors in therapy*: New York: W. W. Norton & Co. .
Courtois, C. A. (1992). The memory retrieval process in incest survivor therapy. *Journal of Child Sexual Abuse, 1,* 15-31. doi:10.1300/J070v01n01_02
Crews, F. (1995). *The memory wars: Freud's legacy in dispute*: New York: New York Review of Books.
Dalenberg, C. (1996). Accuracy, timing and circumstances of disclosure in therapy of recovered and continuous memories of abuse. *Journal of Psychiatry and Law, 24,* 229-275.
Deese, J. (1959). On the prediction of occurrence of particular verbal intrusions in immediate recall. *Journal of Experimental Psychology, 58,* 17-22. doi:10.1037/h0046671
Della Femina, D., Yeager, C. A., & Lewis, D. O. (1990). Child abuse - Adolescent records vs adult recall. *Child Abuse & Neglect, 14*, 227-231.
Elliott, D. M., & Briere, J. (1995). Posttraumatic stress associated with delayed recall of sexual abuse - A general population study. *Journal of Traumatic Stress, 8*, 629-647. doi:10.1007/bf02102892
Enns, C. Z., McNeilly, C. L., Corkery, J. M., & Gilbert, M. S. (1995). The debate about delayed memories of child sexual abuse - A feminist perspective. *Counseling Psychologist, 23*, 181-279. doi:10.1177/0011000095232001
Feldman-Summers, S., & Pope, K. S. (1994). The experience of forgetting child abuse - A national survey of psychologists. *Journal of Consulting and Clinical Psychology, 62,* 636-639. doi:10.1037//0022-006x.62.3.636
Finkelhor, D., Hotaling, G., Lewis, I. A., & Smith, C. (1990). Sexual abuse in a national survey of adult men and women - Prevalence, characteristics, and risk factors. *Child Abuse & Neglect, 14*, 19-28. doi:10.1016/0145-2134(90)90077-7
Fredrickson, R. (1992). *Repressed memories: A journey to recovery from sexual abuse*: New York: Simon and Schuster.
Freyd, J. J. (1996). *Betrayal trauma: The logic of forgetting childhood abuse*: Cambridge, MA: Harvard University Press.
Freyd, J. J., & Gleaves, D. H. (1996). "Remembering" words not presented in lists: Relevance to the current recovered/false memory controversy. *Journal of Experimental Psychology-Learning Memory and Cognition, 22,* 811-813. doi:10.1037/0278-7393.22.3.811
Garry, M., Manning, C. G., Loftus, E. F., & Sherman, S. J. (1996). Imagination inflation: Imagining a childhood event inflates confidence that it occurred. *Psychonomic Bulletin & Review, 3,* 208-214. doi:10.3758/bf03212420
Geraerts, E., Arnold, M. M., Lindsay, D. S., Merckelbach, H., Jelicic, M., & Hauer, B. (2006). Forgetting of prior remembering in persons reporting recovered memories of childhood sexual abuse. *Psychological Science, 17,* 1002-1008. doi:10.1111/j.1467-9280.2006.01819.x
Geraerts, E., Lindsay, D. S., Merckelbach, H., Jelicic, M., Raymaekers, L., Arnold, M. M., & Schooler, J. W. (2009). Cognitive mechanisms underlying recovered-memory experiences of childhood sexual abuse. *Psychological Science, 20,* 92-98. doi:10.1111/j.1467-9280.2008.02247.x
Geraerts, E., Schooler, J. W., Merckelbach, H., Jelicic, M., Hauer, B. J. A., & Ambadar, Z. (2007). The reality of recovered memories - Corroborating continuous and discontinuous memories of childhood sexual abuse. *Psychological Science, 18,* 564-568. doi:10.1111/j.1467-9280.2007.01940.x
Geraerts, E., Smeets, E., Jelicic, M., van Heerden, J., & Merckelbach, H. (2005). Fantasy proneness, but not self-reported trauma is related to DRM performance of women

reporting recovered memories of childhood sexual abuse. *Consciousness and Cognition, 14*, 602-612. doi:10.1016/j.concog.2005.01.006
Gold, S. N., Hughes, D., & Hohnecker, L. (1994). Degrees of repression of sexual abuse memories. *American Psychologist, 49,* 441-442. doi:10.1037/0003-066x.49.5.441
Golding, J. M., Sanchez, R. P., & Sego, S. A. (1996). Do you believe in repressed memories? *Professional Psychology-Research and Practice*, *27*, 429-437. doi:10.1037/0735-7028.27.5.429
Golding, J. M., Sego, S. A., Sanchez, R. P., & Hasemann, D. (1995). The believability of repressed memories. *Law and Human Behavior, 19*, 569-592. doi:10.1007/bf01499375
Harvey, M. R., & Herman, J. L. (1994). Amnesia, partial amnesia, and delayed recall among adult survivors of childhood trauma. *Consciousness and Cognition, 3*, 295-306. doi:10.1006/ccog.1994.1017
Herman, J. L. (1981). *Father-daughter incest*: Cambridge, MA: Harvard University Press.
Herman, J. L. (1992). *Trauma and recovery*: New York: Basic Books.
Herman, J. L., & Schatzow, E. (1987). Recovery and verification of memories of childhood sexual abuse. *Psychoanalytic Psychology, 4,* 1-14.
Holmes, D. S. (1990). The evidence for repression: An examination of sixty years of research. In J. L. Singer (Ed.), *Repression and dissociation: Implications for personality theory, psychopathology, and health*: (pp. 85-102). Chicago, IL: University of Chicago Press.
Hyman, I. E., & Billings, F. J. (1998). Individual differences and the creation of false childhood memories. *Memory, 6,* 1-20. doi:10.1080/741941598
Hyman, I. E., Husband, T. H., & Billings, F. J. (1995). False memories of childhood experiences. *Applied Cognitive Psychology, 9*, 181-197. doi:10.1002/acp.2350090302
Johnson, M. K., Hashtroudi, S., & Lindsay, D. S. (1993). Source monitoring. *Psychological Bulletin, 114,* 3-28. doi:10.1037/0033-2909.114.1.3
Key, H. G., Warren, A. R., & Ross, D. F. (1996). Perceptions of repressed memories: A reappraisal. *Law and Human Behavior, 20,* 555-563. doi:10.1007/bf01499041
Laurence, J. R., & Perry, C. (1983). Hypnotically created memory among highly hypnotizable subjects. *Science, 222*, 523-524. doi:10.1126/science.6623094
Lief, H. I., & Fetkewicz, J. (1995). Retractors of false memories: The evolution of pseudomemories. *Journal of Psychiatry and Law,* 411-435.
Lindsay, D. S., & Read, J. D. (1994). Psychotherapy and memories of childhood abuse - A cognitive perspective. *Applied Cognitive Psychology, 8*, 281-338. doi:10.1002/acp.2350080403
Lindsay, D. S., & Read, J. D. (1995). "Memory work" and recovered memories of childhood sexual abuse: Scientific evidence and public, professional, and personal issues. *Psychology Public Policy and Law, 1*, 846-908. doi:10.1037/1076-8971.1.4.846
Loftus, E. F. (1979). *Eyewitness testimony*: Cambridge, MA: Harvard University Press.
Loftus, E. F. (1993). The reality of repressed memories. *American Psychologist, 48*, 518-537. doi:10.1037/0003-066x.48.5.518
Loftus, E. F., Garry, M., & Feldman, J. (1994). Forgetting sexual trauma - What does it mean when 38 percent forget? *Journal of Consulting and Clinical Psychology, 62*, 1177-1181. doi:10.1037/0022-006x.62.6.1177
Loftus, E. F., & Ketcham, K. (1994). *The myth of repressed memory*: New York: St. Martin's Press.
Loftus, E. F., & Pickrell, J. E. (1995). The formation of false memories. *Psychiatric Annals, 25,* 720-725. doi:10.3928/0048-5713-19951201-07

Loftus, E. F., Polonsky, S., & Fullilove, M. T. (1994). Memories of childhood sexual abuse - Remembering and forgetting. *Psychology of Women Quarterly, 18*, 67-84. doi:10.1111/j.1471-6402.1994.tb00297.x

Loftus, E. F., Weingardt, K. R., & Hoffman, H. G. (1993). Sleeping memories on trial: Reactions to memories that were previously repressed. *Expert Evidence, 2,* 51-59.

Masson, J. M. (1984). *The assault on truth: Freud's suppression of the seduction theory*: New York, Farrar, Straus & Giroux.

McNally, R. J. (2003). *Remembering trauma*: Cambridge, MA: Harvard University Press.

McNally, R. J., Clancy, S., Barrett, H., & Parker, H. (2004). Inhibiting retrieval of trauma cues in adults reporting histories of childhood sexual abuse. *Cognition & Emotion, 18,* 479-493. doi:10.1080/02699930341000400

McNally, R. J., Clancy, S. A., Barrett, H. M., & Parker, H. A. (2005). Reality monitoring in adults reporting repressed, recovered, or continuous memories of childhood sexual abuse. *Journal of Abnormal Psychology, 114,* 147-152. doi:10.1037/0021-843x.114.1.147

McNally, R. J., Clancy, S. A., & Schacter, D. L. (2001). Directed forgetting of trauma cues in adults reporting repressed or recovered memories of childhood sexual abuse. *Journal of Abnormal Psychology, 110*, 151-156. doi:10.1037/0021-843x.110.1.151

McNally, R. J., Clancy, S. A., Schacter, D. L., & Pitman, R. K. (2000). Personality profiles, dissociation, and absorption in women reporting repressed, recovered, or continuous memories of childhood sexual abuse. *Journal of Consulting and Clinical Psychology, 68,* 1033-1037. doi:10.1037/0022-006x.68.6.1033

McNally, R. J., & Geraerts, E. (2009). A new solution to the recovered memory debate. *Perspectives on Psychological Science, 4*, 126-134. doi:10.1111/j.1745-6924.2009.01112.x

Myers, L. B., Brewin, C. R., & Power, M. J. (1998). Repressive coping and the directed forgetting of emotional material. *Journal of Abnormal Psychology, 107*, 141-148. doi:10.1037/0021-843x.107.1.141

Neisser, U., & Harsch, N. (1992). *Phantom flashbulbs - False recollections of hearing the news about Challenger* (Vol. 4).

Ofshe, R., & Watters, E. (1994). *Making monsters: False mmeories, psychotherapy, and sexual hysteria*: Berkeley, CA: University of California Press.

Ofshe, R. J. (1992). Inadvertent hypnosis during interrogation - False confession due to dissociative state - Misidentified multiple personality and the satanic cult hypothesis. *International Journal of Clinical and Experimental Hypnosis, 40*, 125-156. doi:10.1080/00207149208409653

Pendergrast, M. (1995). *Victims of memory: Incest accusations and shattered lives*: Hinesburg VT: Upper Access.

Pezdek, K., Finger, K., & Hedge, D. (1997). Planting false childhood memories: The role of event plausibility. *Psychological Science, 8,* 437-441. doi:10.1111/j.1467-9280.1997.tb00457.x

Polusny, M. A., & Follette, V. M. (1996). Remembering childhood sexual abuse: A national survey of psychologists' clinical practices, beliefs, and personal experiences. *Professional Psychology-Research and Practice, 27*, 41-52. doi:10.1037/0735-7028.27.1.41

Poole, D. A., Lindsay, D. S., Memon, A., & Bull, R. (1995). Psychotherapy and the recovery of memories of childhood sexual abuse - US and British practitioners' opinions, practices, and experiences. *Journal of Consulting and Clinical Psychology, 63*, 426-437. doi:10.1037/0022-006x.63.3.426

Pope, H. G., & Hudson, J. I. (1995). Can memories of childhood abuse be repressed? *Psychological Medicine, 25*, 121-126. doi:10.1017/s0033291700028142

Pope, K. S. (1996). Memory, abuse, and science - Questioning claims about the false memory syndrome epidemic. *American Psychologist, 51*, 957-974.

Pope, K. S., & Brown, L. S. (1996). *Recovered memories of abuse: Assessment, therapy, forensics*: Washington, DC: American Psychological Association.

Porter, S., Yuille, J. C., & Lehman, D. R. (1999). The nature of real, implanted, and fabricated memories for emotional childhood events: Implications for the recovered memory debate. *Law and Human Behavior, 23*, 517-537. doi:10.1023/a:1022344128649

Pynoos, R. S., & Nader, K. (1989). Children's memory and proximity to violence. *Journal of the American Academy of Child and Adolescent Psychiatry, 28*, 236-241. doi:10.1097/00004583-198903000-00015

Roediger, H. L., & McDermott, K. B. (1995). Creating false memories - Remembering words not presented in lists. *Journal of Experimental Psychology-Learning Memory and Cognition, 21*, 803-814. doi:10.1037/0278-7393.21.4.803

Schacter, D. L. (1996). *Searching for memory: The brain, the mind and the past*. New York: Basic Books.

Schooler, J. W., Ambadar, Z., & Bendiksen, M. S. (1997). A cognitive corroborative case study approach for investigating discovered memories of sexual abuse. In J. D. Read & D. S. Lindsay (Eds.), *Recollections of Trauma: Scientific Evidence and Clinical Practice* (Vol. 291, pp. 379-387).

Schooler, J. W., Bendiksen, M., & Ambadar, Z. (1997). Taking the middle line: Can we accommodate both fabricated and recovered memories of sexual abuse? In M. A. Conway (Ed.), *Recovered memories and false memories* (pp. 251-292). Oxford: Oxford University Press.

Spence, D. P. (1982). *Narrative truth and historical truth: Meaning and interpretation in psychoanalysis*: New York: Norton.

Terr, L. (1988). What happens to early memories of trauma - A study of 20 children under age 5 at the the time of documented traumatic events. *Journal of the American Academy of Child and Adolescent Psychiatry, 27*, 96-104. doi:10.1097/00004583-198801000-00015

Terr, L. (1994). *Unchained memories: True stories of traumatic memories, lost and found*: New York: Basic Books.

Terr, L. C. (1991). Childhood traumas - An outline and overview. *American Journal of Psychiatry, 148*, 10-20.

van der Kolk, B. A. (1994). The body keeps the score: memory and the evolving psychobiology of posttraumatic stress. *Harvard Review of Psychiatry, 1*, 253-265. doi:10.3109/10673229409017088

van der Kolk, B. A., & Fisler, R. (1995). Dissociation and the fragmentary nature of traumatic memories - Overview and exploratory study. *Journal of Traumatic Stress, 8*, 505-525. doi:10.1002/jts.2490080402

Wakefield, H., & Underwager, R. (1992). Recovered memories of alleged sexual abuse - Lawsuits against parents. *Behavioral Sciences & the Law, 10*, 483-507. doi:10.1002/bsl.2370100406

Williams, L. M. (1994). Recall of childhood trauma - A prospective study of women's memories of child sexual abuse. *Journal of Consulting and Clinical Psychology, 62*, 1167-1176. doi:10.1037//0022-006x.63.3.343

Williams, L. M. (1995). Recovered memories of abuse in women with documented child sexual victimization histories. *Journal of Traumatic Stress*, *8*, 649-673. doi:10.1007/bf02102893

Yapko, M. D. (1994a). Suggestibility and repressed memories of abuse - A survey of psychotherapists' beliefs. *American Journal of Clinical Hypnosis, 36*, 163-171. doi:10.1080/00029157.1994.10403066

Yapko, M. D. (1994b). *Suggestions of abuse: True and false memories of childhood sexual trauma*: New York: Simon & Schuster.
